# Retraction Note to: LncRNA BCRT1 promotes breast cancer progression by targeting miR-1303/PTBP3 axis

**DOI:** 10.1186/s12943-022-01576-y

**Published:** 2022-06-17

**Authors:** Yiran Liang, Xiaojin Song, Yaming Li, Bing Chen, Wenjing Zhao, Lijuan Wang, Hanwen Zhang, Ying Liu, Dianwen Han, Ning Zhang, Tingting Ma, Yajie Wang, Fangzhou Ye, Dan Luo, Xiaoyan Li, Qifeng Yang

**Affiliations:** 1 Department of Breast Surgery, Qilu Hospital of Shandong University, Jinan, Shandong 250012 People’s Republic of China; 2 Pathology Tissue Bank, Qilu Hospital of Shandong University, Jinan, Shandong 250012 People’s Republic of China

**Retraction note to: Mol Cancer 19, 85 (2020)**

**https://doi.org/10.1186/s12943-020-01206-5**

The Editor-in-Chief has retracted this article. After publication, the authors alerted the journal that there were errors in Figs. 3b, 4j, S2g and S2h, resulting in overlap between images representing different treatment groups. The Editor-in-Chief therefore no longer has confidence in the presented data.

Yiran Liang, Xiaojin Song, Yaming Li, Bing Chen, Wenjing Zhao, Lijuan Wang, Hanwen Zhang, Ying Liu, Dianwen Han, Ning Zhang, Yajie Wang, Fangzhou Ye, Dan Luo, Xiaoyan Li and Qifeng Yang agree to this retraction. Tingting Ma has not responded to any correspondence from the editor or publisher about this retraction.

